# Frequency of functional exonic single-nucleotide polymorphisms and haplotype distribution in the *SLCO1B1* gene across genetic ancestry groups in the Qatari population

**DOI:** 10.1038/s41598-022-19318-x

**Published:** 2022-09-01

**Authors:** Mohammed Dashti, Abdullah Al-Matrouk, Arshad Channanath, Fahd Al-Mulla, Thangavel Alphonse Thanaraj

**Affiliations:** 1 Genetics and Bioinformatics Department, Dasman Diabetes Institute, Kuwait City, Kuwait; 2 Narcotic and Psychotropic Department, Ministry of Interior, Farwaniya, Kuwait

**Keywords:** Genetics, Risk factors

## Abstract

Organic anion transporting polypeptides (OATP), which are encoded by SLCO genes, participate in the hepatic elimination of drugs and xenobiotics. *SLCO1B1* is an important pharmacogenomic gene (encoding OATP1B1) associated with response to the uptake of endogenous compounds, such as statin and bilirubin. Ethnicity of the patient modulates the response to these drugs; the frequency and haplotype data for *SLCO1B1* genetic variants in the Arab population is lacking. Therefore, we determined the frequencies of two well-characterized *SLCO1B1* single nucleotide polymorphisms (SNP) and haplotypes that affect the OATP1B1 drugs transportation activity in Qatari population. Genotyping data for two *SLCO1B1* SNPs (c.388A > G, c.521 T > C) were extracted from whole exome data of 1050 Qatari individuals, who were divided into three ancestry groups, namely Bedouins, Persians/South Asians, and Africans. By way of using Fisher's exact and Chi-square tests, we evaluated the differences in minor allele frequency (MAF) of the two functional SNPs and haplotype frequencies (HF) among the three ancestry groups. The OATP1B1 phenotypes were assigned according to their function by following the guidelines from the Clinical Pharmacogenetics Implementation Consortium for *SLCO1B1* and Simvastatin-Induced Myopathy.The MAF of *SLCO1B1*:c.388A > G was higher compared to that of *SLCO1B1*:c.521 T > C in the study cohort. It was significantly high in the African ancestry group compared with the other two groups, whereas *SLCO1B1*:c.521 T > C was significantly low in the African ancestry group compared with the other two groups. The *SLCO1B1* *15 haplotype had the highest HF, followed by *1b, *1a, and *5. Only the *SLCO1B1* *5 haplotype showed no significant difference in frequency across the three ancestry groups. Furthermore, we observed that the OATP1B1 normal function phenotype accounted for 58% of the Qatari individuals, the intermediate function phenotype accounted for 35% with significant differences across the ancestry groups, and the low function phenotype accounted for 6% of the total Qatari individuals with a higher trend observed in the Bedouin group.The results indicate that the phenotype frequencies of the OATP1B1 intermediate and low function in the Qatari population appear at the higher end of the frequency range seen worldwide. Thus, a pharmacogenetic screening program for *SLCO1B1* variants may be necessary for the Qatari population.

## Introduction

Transmembrane protein transporters are involved in the flow of ions, small molecules, and macromolecules across the cell membrane. There are two main groups of proteins known to be involved in the transportation process, namely (a) the solute-linked carrier (SLC) superfamily (influx transporters), and (b) the ATP-binding cassette (ABC) superfamily (efflux transporters)^1^. The organic anion transporting polypeptide (OATP) transporters, which are expressed on the sinusoidal membrane of human hepatocytes, belong to the SLC superfamily and play an important role in the hepatic elimination of drugs and xenobiotics^2^. There are 11 members in the human OATP family that are encoded by the *SLCO* genes. The OATP1B1, that is encoded by *SLCO1B1*, is the most fully characterized human OATP and is highly polymorphic^3^.

Single nucleotide polymorphisms (SNP) of the *SLCO1B1* gene can alter OATP1B1 activity in vitro and in vivo, and can affect the pharmacokinetics and pharmacodynamics of various drugs^4–8^. OATP1B1 mediates the uptake of many endogenous substrates and xenobiotics including bilirubin, bile acids, bile salt, thyroid hormones, steroid conjugates of cyclic peptides, antibiotics (such as rifampicin and benzylpenicillin), methotrexate rheumatic arthritis, and natural toxins (such as microcystin and phalloidin)^9,10^. In addition, OATP1B1 is important for mediating the hepatic clearance of drugs such as 3-hydroxy-3-methylglutaryl coenzyme A (HMG-CoA) reductase inhibitors (*e.g.* statins: pravastatin and simvastatin), angiotensin-converting enzyme inhibitors (enalapril), antibiotics (rifampicin), chemotherapeutic agents, endothelin receptor antagonist (bosentan, prostacyclin, and beraprost) anti-inflammatory drug (diclofenac), and antiviral agent (simeprevir)^11–15^.

The two well-characterized functional *SLCO1B1* variants are SLCO1B1*1b (c.388A > G rs2306283) and SLCO1B1*5 (c.521 T > C rs4149056). While the c.388A > G variant is associated with increased OATP1B1 activity, the c.521 T > C variant is associated with reduced transport activity^6,16^. Decreased hepatic drug clearance is seen in carriers of SLCO1B1*5 variant^17^. An increase in the transport of pravastatin and ezetimibe has been reported in carriers of SLCO1B1*1b variant in different populations^18–20^. In contrast, reduced uptake of statins, fexofenadine, irinotecan, and repaglinide into hepatocytes is associated with the SLCO1B1*5 polymorphism^21–26^. The SLCO1B1*5 variant is associated with higher plasma concentrations of lopinavir, which is an antiretroviral protease inhibitor used to treat HIV infections^27,28^.

Many global studies have examined alterations in OATP1B1 transporter activity associated with SNPs from *SLCO1B1* in different populations including European^29^, European American, African American^8^, Asian^3^, and North African^18^; however, there is none or limited data from Arab populations. Arab populations are characterized by a high rate of consanguineous marriages and by a high prevalence of metabolic disorders. In particular, there is a high prevalence of dyslipidaemia, which is a contributing factor to the increase in the cardiovascular disease (CVD)-associated mortality rate in Middle East^30^. Given that dyslipidaemia is managed by anionic drug that transported OATP1B1, lack of studies from Middle East on pharmacogenomics of OATP1B1 is a concern. Thus, the focus of the current study is to characterize the two well-known SNPs of *SLCO1B1* and determine their frequencies in the Qatari population. In addition, pharmacogenetic studies show that there are implications from racial and ancestry variations within the same population on defining genetic profiles for personalised medicine^31^. Therefore, the current study also examined the three genetic substructures within the Qatari population, namely Bedouin/Arab, Persian/South Asian, and African ancestry groups^32^.

## Results

### Minor allele frequencies (MAF) of the two exonic SNPs from the *SLCO1B1* gene in the study cohort

We calculated minor allele frequency (MAF) and Hardy–Weinberg equilibrium for the study variants. The study variants passed the tests for Hardy–Weinberg equilibrium (HWE). The MAF for the two exonic SNPs of the *SLCO1B1* gene and the HF of *SLCO1B1* *1a, *1b, *5, and *15 for the 1050 Qatari individuals across the three ancestry groups are presented in Table 1. The MAF of the *SLCO1B1*:c.388A > G was significantly higher than that of *SLCO1B1*:c.521 T > C (*p* < 0.001) in the full cohort of 1050 Qatari individuals and in each of the three ancestry groups. The MAF of *SLCO1B1*:c.388A > G was significantly higher (*p* < 0.001) in the African ancestry group compared with the other two groups, which exhibited similar frequency values. The MAF of *SLCO1B1*:c.521 T > C was significantly lower (*p* = 0.007) in the African ancestry group compared with the other two groups, which had similar frequency values.

**Table 1:** Minor allele and haplotype frequencies of *SLCO1B1* functional SNPs in Qatar across the three different ancestry groups.

*SLCO1B1*	Total	Bedouin/Arab	Persian/South Asian	African	*p* value (statistical significance of the MAF differences across the three ancestral groups)
**Minor allele (Second most common allele) frequencies**					
**N = count of study participants**	**1050**	**587**	**387**	**76**	
c.388A > G G allele	0.49	0.49	0.48	0.61	0.0107
c.521 T > C C allele	0.24	0.27	0.22	0.14	0.0003
**Haplotype (Combination of alleles present across multiple loci on single chromosome) frequencies (%)**					
**N**	**2,100**	**1174**	**774**	**152**	
*1a^a^	23.24	23.68	24.55	13.16	0.0085
*1b^b^	34.95	29.30	37.73	64.47	< 0.001
*5^c^	3.81	3.58	4.65	1.32	0.1195
*15^d^	38.00	43.44	33.07	21.05	< 0.001

Significant value are in [bold].

a = wild type at all loci; b = rs2306283 G allele (A ancestral) (c.388A > G; p.N130D); c = rs4149056 C allele (T ancestral) (c.521 T > C; p.V174A); d = rs2306283 G allele (A ancestral) and rs4149056 C allele (T ancestral).

### Haplotype frequencies (HF) of the exonic SNPs from the *SLCO1B1* gene

Table 1 shows that the *SLCO1B1* *15 haplotype exhibited the highest frequency followed by *1b, *1a, and *5. Only the *SLCO1B1* *5 haplotype showed no significant difference in frequencies across the three ancestry groups (*p* = 0.1195). The frequency of the *SLCO1B1* *1a was significantly lower in the African group and that of *1b was significantly higher in the African group. The frequency of the *SLCO1B1* *15 haplotype was significantly higher in the Bedouin/Arab group when compared with those in the other two groups. The frequency of the *5 haplotype was as such as small in the whole of the cohort.

### Frequency of the OATP1B1 phenotypes

The frequency of the normal function phenotype of the OATP1B1 transporter was highest among the whole cohort (at 58%) while that of the low function was lowest (at 6%). Among the three ancestral groups, the African group exhibited higher frequency for the normal function phenotype while the Bedouin group exhibited higher frequency for the intermediate function phenotype.

### Minor allele frequencies (MAF) of the two exonic *SLCO1B1* SNPs in global populations

Results of our survey of literature and genotype databases for allele frequencies at the two *SLCO1B1* SNPs across different global populations are presented in Table 2. The frequencies exhibit considerable variation among the populations.

**Table 2:** Allele frequency values for the two functional exonic variants from *SLCO1B1* in different global populations.

	Allele frequency for c.388A > G	Allele frequency for c.521 T > C	References
Qatari	0.49	0.24	This study
Thai	0.78	0.12	^51^
Koreans	0.75	0.25	^46^
Han Chinese	0.15	0.16	^53^
	0.73	0.14	^59^
Chinese	0.73	0.11	^60^
	0.80	0.13	^3^
	0.67	0.086	^61^
	0.71	0.11	^62^
Japanese	0.63	0.16	^26^
	0.74	0.19	^60^
African Americans	0.75	0.023	^8^
European Americans	0.72	0.13	^53^
	0.30	0.14	^8^
Europeans	0.40	0.16	gnomAD
	0.41	0.18	^60^
Israeli	0.46	0.20	^60^
Pakistani	0.47	0.09	^60^
Caucasians	0.37	0.15	^16^
Finnish	0.45	0.21	gnomAD
	0.46	0.20	^29^
Turkish	0.46	0.12	^18^
Macedonians	0.41	0.14	^63^
Albanians	0.42	0.12	^63^
Greeks	0.43	0.16	^64^
American	0.43	0.11	gnomAD
Ashkenazi Jewish	0.45	0.18	gnomAD
Non-finish Europeans	0.40	0.15	gnomAD
South Asians	0.47	0.05	gnomAD
Dutch	–	0.18	^65^
Algerians	0.64	0.17	^60^
Sub-Saharan African	0.79	0.019	^60^
Zulu (South Africa)	0.009	0	^31^
Cape Admixture (South Africa)	0	0.60	^31^

## Discussion

OATP1B1 (encoded by *SLCO1B1*) is a well-characterized human OATP and it is highly polymorphic^3,8^. The genetic variations of the *SLCO1B1* gene have been documented for various populations^3,8,18,29^; however, there is a lack of data regarding these variations in the Arabian countries, including the Gulf Cooperation Council countries. Therefore, we evaluated the genetic variations in the *SLCO1B1* among the Qatari population which is comprised of three ethnic groups, namely Bedouins/Arabs, Persians/South Asians, and Africans^32^. In total, 1050 individuals were examined in this study in which 587 (55.9%) were of Bedouin/Arab ancestry, 387 (36.8%) were of Persian/South Asian ancestry, and 76 (7.2%) were of African ancestry.

Considering that the *SLCO1B1* *1b, *5, and *15 are the most-often implicated variants in altering the function and/or intracellular disposition of OATP1B1 substrates^14^, we focused on these variants in the Qatari population. Previous studies have demonstrated that carriers of the *1b allele exhibit increased transport activity of pravastatin and decreased plasma concentrations of ezetimibe^19,20^, whereas carriers of the *5 variant show reduced uptake of statins, including pravastatin^26,33^ and rifampicin^34^, in hepatocytes, as well as show increased area under the AUC curve for fexofenadine, repaglinide, and irinotecan^21,23–25^. Furthermore, SLCO1B1*5 is strongly associated with myopathy among simvastatin users^1,35–45^.

The *15 haplotype was shown to be associated with increased plasma concentrations of pitavastatin in Korean subjects^46^ and had an effect on the systemic exposure and elimination of atorvastatin^47–49^. Moreover, *15 carriers showed a reduction in transport activity and this haplotype was associated with myopathy in patients administered with pravastatin and atorvastatin1^36,38,45,50^.

By way of considering data from literature^51–53^ as well as data extracted from the 1000 Genomes project and gnomAD databases, we compared the minor allele frequencies of the variants from Qatari population with those from global populations. The allele frequency of the c.388A > G variant in the Qatari populations had no similarities with that from other documented populations including Thai, Koreans, Han Chinese, Japanese, African Americans, and European Americans (see Table 2). However, the European, Israeli, Pakistani, Caucasian, Finnish, Turkish, Macedonian, Albanian, and Greek populations exhibited a very similar allele frequency. Data obtained from the gnomAD database also showed a close similarity in allele frequencies from the combined-European, American, Ashkenazi Jewish, Finnish, non-Finish European, and South Asian populations. Moreover, the MAF of c.388A > G in these populations was also similar to that from the Persian/South Asian and Bedouin/Arab ethnic groups from Qatar.

The non-synonymous c.521 T > C SNP, which is associated with reduced OATP1B1 activity, was found in the Qatari population with an allele frequency dissimilar to that of Macedonian, Albanian, Thai, Caucasian, Dutch, Finnish, Algerian, Israeli, Sub-Saharan African, Japanese, Korean, Chinese, African American, European American, Han Chinese, and Turkish populations. However, the frequency from the African ethnic group was similar to that from the European American, Japanese, Chinese, Korean, Thai, and Finnish populations (see Table 2).

Thus, it appears that the minor allele frequencies of both the variants from the Qatari population were dissimilar to those from many Asian countries, particularly the East Asian Countries.

With regard to haplotype frequencies, our data points out that the haplotype frequency for the *5 haplotype in Qataris (HF = 3.81%) is similar to that in the Middle Eastern population (HF = 5%), but higher than that seen in other populations including Thai, Asians, South/central Asians and Indians, Oceania, South/Central Americans, Africans (HF = 0%), and Caucasians (HF = 1%)^51^. With respect to the Qatari ethnic groups, the frequency of the *5 haplotype in Bedouin/Arab ethnic group (HF = 3.58%) and Persian ethnic group (HF = 4.65%) was also similar to that seen in the Middle Eastern population, whereas that seen in the Persian population was much higher compared with that from the South/Central Asians and Indians (SW Asians) (HF = 0%)^51^. In addition, the frequency seen in the African ethnic group of Qatar (HF = 1.32%) was higher when compared to that from the natives of Africa^51^. Regarding the frequency of the *15 haplotype, our results indicated that there are no similarities with global populations, except for the African ethnic group (HF = 21.05%) with the South/Central Americans (HF = 24%)^51^.

When comparing the frequency of the *1b haplotype from the Qatari population (HF = 34.24%) with those from global populations, similarities were observed with the Middle Eastern (HF = 31%) and South/Central American (HF = 39%) populations. The haplotype frequency of *1b was comparable between the African ethnic group from Qatar (HF = 64.47%) and the Thai (HF = 65%) and Oceania (HF = 66%) populations^51^.

Thus, it appears that the *SLCO1B1* HFs from Qatar are similar to those seen in Middle Eastern population and that the results from Qatar are extendable to the whole of Middle Eastern countries. In summary, the *SLCO1B1* HFs in Qataris are unique from global populations.

As regards *SLCO1B1* diplotypes, the phenotype frequency (PF) of the OATP1B1 transporter accounts for 58.19% with normal function and 35.33% for intermediate function (see Table 3), These phenotype frequencies of the Qatari population are within the global PF range (55–88% and 11–36%, respectively). Compared with other populations (including the Thai, Koreans, Chinese, and Vietnamese) both the intermediate and normal function-encoding diplotypes were higher in Qatar^51,52^. However, the phenotype frequency of the *SLCO1B1* diplotypes encoding low function OATP1B1 was higher in Qatar than the global range (0–6%), whereas the PF values for all of the ethnic groups of normal and intermediate function were within the global range. Hence, it can be concluded that the phenotypic frequency of the intermediate and the low OATP1B1 function phenotypes in the Qatari population appear at either the top end of the global range or even higher. This warrants a large-level pharmacogenetic screening of the Qatari population for *SLCO1B1* variants to guide clinical decisions regarding the above-mentioned drugs and xenobiotics (see the “Introduction” section).

**Table 3:** OATP1B1 phenotypes based on *SLCO1B1* diplotypes in Qatar across the three different ancestry groups.

OATP1B1 phenotypes and genotype definition	*SLCO1B1* diplotypes (Haplotype pairs on homologous chromosomes)	Phenotype frequencies (%)				*p* value
		Total	Bedouin/Arab	Persian/South Asian	African	
Normal function (Individuals carrying two functional alleles)	*1a/*1a	58.19	52.98	62.27	77.63	< 0.001
	*1a/*1b					
	*1b/*1b					
Intermediate function (Individuals carrying one reduced-function alleles)	*1a/*5	35.33	39.69	32.3	17.11	< 0.001
	*1a/*15					
	*1b/*5					
	*1b/*15					
Low function (Individuals carrying two reduced-function alleles)	*5/*5	6.48	7.33	5.43	5.26	0.488
	*5/*15					
	*15/*15					

Further, it is reiterated that the OATP1B1 transporter intermediate function has a significant variability across the three Qatari ethnic groups—this observation is important to consider in personalized medicine approach where the genomic sequence of every patient determines the best therapy and dosage regimen suited to the patient. The same conclusion has been drawn in a previous study that investigated South African ethnic populations including Zulu and Cape admixture groups^31^.

The current study is limited to only two functional SNPs from *SLCO1B1* that are used to clinically classify/predict OTAB1 phenotype profile. However, it is to be noted that this is the first study of pharmacogenomic aspects of *SLCO1B1* variations in Arab populations and it is important to restrict the study to only the variants that are globally well-characterized for clinical outcome following the use of drugs. Nevertheless, we hope that our work will pave the way to follow-up works by other researchers on patients to study the impact of SNPs on key transporter proteins, which may hinder drug efficacy in vitro, in vivo, and clinical setting.

## Conclusion

Based on the observations from our study, we predict that the Qatari population, along with the different ethnic groups of the Bedouin/Arab ancestry, Persian/South Asian ancestry, and African ancestry, is at an increased risk of haplotype-mediated inter-individual variations in drug disposition and, therefore, is prone to elevated toxicity levels compared with other populations. However, since there is no previously published data on association of these variants in the Middle Eastern population, particularly the Qatari population, in a clinical setting, we highly recommend that a pharmacogenetic screen should be conducted on a large scale for this population in a clinical setting.

## Materials and methods

### Ethics statement

This study was approved by the Institutional Ethical Review Committee at the Dasman Diabetes Institute, Kuwait, in accordance with the Declaration of Helsinki. Genomic information on the study subjects is publicly available from the National Center for Biotechnology Information Sequence Read Archive (NCBI-SRA). Informed consent from the recruited participants had been obtained as part of the original studies^32,54^ using protocols approved by the institutional review boards of the Hamad Medical Corporation and Weill Cornell Medical College in Qatar.

### Study samples

Next-generation sequence data of whole exomes and genomes from individuals living in Qatar^32,54^ are publicly available at the NCBI-SRA server (accession numbers SRP060765, SRP061943, and SRP061463). These exomes and genomes were sequenced on the Illumina platform with library kits of differing coverage of the human consensus-coding sequence regions. Further, some of these individuals were not natives of Qatar and some of them were of ad-mixed ethnicities. To address these concerns, we short-listed only those native individuals who adhered to one of the three ancestry-components of Qatar, namely Bedouins, Persian/South Asians and Africans^32,54^. Further, we ignored those individuals who were sequenced on low-coverage library kits, such as Agilent version V4 (Agilent Technologies Inc., USA). This led to the short-listed sample set of 958 exomes and 92 genomes.

Thus, the study sample set included a total of 1050 individuals, of which 449 individuals were males (43%) and 601 were females (57%).

### Variant calling for the *SLCO1B1* gene

Illumina raw paired-end reads from the whole genome and exome sequencing were mapped to the human genome reference assembly, GRCh37, by the Burrows–Wheeler Aligner version v07-17^55^. The mapped data were converted to BAM files in which duplicate reads were removed using SAMtools^56^ and Picard software tool version 2.20.21 (http://picard.sourceforge.net). The Genome Analysis Toolkit (GATK) version v3.8-1-0^57^ was used for local realignment of the reads around insertion–deletion mutations, for recalibration of base quality, and for variant calling for the *SLCO1B1* variants (rs2306283; c.388A > G and rs4149056; c.521 T > C).

### OATP1B1 transport phenotypes assignment with *SLCO1B1* genotypes

The assignment of the OATP1B1 phenotypes into three groups of normal, intermediate, and low function was done by following the guidelines of the Clinical Pharmacogenetics Implementation Consortium for *SLCO1B1* and Simvastatin-Induced Myopathy: 2014 update and the star (*) allele nomenclature^58^.

### Statistical analysis

The frequency of the two functional SNPs within the *SCLO1B1* (c.388A > G, c.521 T > C) gene was assessed for deviation from the Hardy–Weinberg equilibrium (HWE) using R software (version 3.6.2) (https://www.R-project.org/). Differences in minor alleles frequencies and haplotype frequencies across the three Qatari ancestry groups were characterized using Fisher's exact and Chi-square tests using IBM SPSS Statistics Version 25 software. A *p*-value of < 0.05 was considered statistically significant.

## Data Availability

The next-generation sequencing data used in this study are available from the NCBI-SRA with accession numbers SRP060765, SRP061943, and SRP061463.
